# Effect of red light on epidermal proliferation and mitochondrial activity

**DOI:** 10.1111/srt.13447

**Published:** 2023-08-28

**Authors:** Yuki Umino, Mitsuhiro Denda

**Affiliations:** ^1^ MIRAI Technology Institute Shiseido Co., Ltd Yokohama Japan; ^2^ Meiji University Institute for Advanced Study of Mathematical Sciences Tokyo Japan

**Keywords:** human keratinocytes, mitchondrial activity, proliferation, red light

## Abstract

**Background/Purpose:**

We previously demonstrated that irradiation with red light accelerates recovery of the epidermal water‐impermeable barrier, whereas blue light delays it, and white and green light have no effect. Here, we aimed to examine in detail the effects of red and blue light in a human epidermal‐equivalent model and in human skin.

**Methods:**

We used light‐emitting diodes (red light, 630 nm, 6.2 mW/cm^2^; blue light, 463 nm, 6.2 mW/cm^2^) for irradiation of an epidermal‐equivalent model and human skin. Cell proliferation was evaluated by means of BrdU and Ki‐67 staining, and mitochondrial activity was quantified with an extracellular flux analyzer.

**Results:**

Irradiation of the epidermal‐equivalent model with red light for 2 h (44.64 J/cm^2^) increased both epidermal proliferation in the basal layer and mitochondrial activity. Blue light had no effect on epidermal proliferation. Furthermore, irradiation with red light for 2 h on three consecutive days increased epidermal proliferation in human skin tissue in culture.

**Conclusion:**

These results suggest that red light accelerates epidermal proliferation in both an epidermal‐equivalent model and human skin, and may promote epidermal homeostasis.

## 1 INTRODUCTION

Epidermal barrier homeostasis is influenced by external environmental factors such as light, temperature, electrical potential, etc.^1^ Among them, ultraviolet light is well known to damage the skin.^2^ More recently, effects of blue light and infrared light were also reported.^3^, ^4^, ^5^, ^6^, ^7^ For example, blue light induces oxidative stress.^3^, ^4^ Furthermore, infrared light alone, or in combination with ultraviolet light/visible light/heat was reported to suppress keratinocyte proliferation,^5^ induce reactive oxygen species (ROS) in keratinocytes and fibroblasts,^6^ increase matrix metalloproteinase 1 (MMP‐1) and decrease type I procollagen in human skin.^7^ In addition, red light has various effects on the proliferation, viability, or migration of epidermal keratinocytes,^8^, ^9^, ^10^, ^11^ depending upon the intensity and duration of irradiation.

We previously demonstrated that epidermal barrier recovery was accelerated after irradiation with red light, while it was delayed after irradiation with blue light, and was unaffected by white or green light.^12^ Red light also accelerated lamellar body secretion between the stratum corneum and stratum granulosum, while blue light decreased it, as assessed by electron microscopy. In addition, Abe et al., reported that red light promoted barrier recovery and blue light had no effect, based on measurements of transepidermal potential.^13^

The aim of the present work was to examine and compare the effects of red and blue light on epidermal proliferation in a human epidermal‐equivalent model^14^, ^15^, ^16^ and in human skin, and also to evaluate the effects on mitochondrial activity, which is deeply related to cell proliferation, differentiation and epidermal homeostasis.^17^

## 2 MATERIALS AND METHODS

### 2.1 Construction of the epidermal equivalent model

The protocol for construction of the epidermal‐equivalent model was described previously.^14^, ^15^, ^16^ Normal human keratinocytes (NHEKs) from Kurabo (Osaka, Japan) were cultured in Epilife‐KG2 (Kurabo) containing 0.06 mM Ca^2+^. Millicells with hanging 0.4 μm PET inserts (Millipore, Billerica, MA) were incubated with CellStart (Invitrogen Life Technologies, Carlsbad, CA) in 50X DPBS dilution. Then, 2.2 × 10^5^ keratinocytes/500 μL in CnT Prime media (CELLnTEC, Bern, Switzerland) were seeded and CnT Prime medium was added outside the inserts. After 3 days, the medium was removed and CnT‐PR‐3D differentiation medium (CELLnTEC) containing 50 μg/mL ascorbic acid was added both inside and outside the inserts. On the next day, cultures were lifted to the air‐medium interface by removing the medium inside the inserts and reducing the amount outside the inserts to 500 μL. Cultures were fed every day with 500 μL of differentiation medium.

### 2.2 Red and blue light irradiation and fixation

Irradiation was conducted with arrays of 60 light‐emitting diodes (LED, LP‐FPC100C50‐30R‐W/R‐WP, Peace Corporation Co., Ltd., Koshigaya, Saitama, Japan) at 6.2 mW/cm^2^ (red light peak wavelength 630 nm, half bandwidth 17 nm; blue light peak wavelength 463 nm, half bandwidth 20 nm; see Figure S1). Spectra were measured with a QE Pro Spectrometer (Ocean Photonics, Tokyo, Japan). On day 13, the epidermal‐equivalent model was irradiated with red or blue light for a designated time. After exposure, culture media were changed and cells were stained with 10 μM 5‐bromo‐2′‐deoxyuridine (BrdU) in differentiation medium. On the next day, the models were fixed with 4% paraformaldehyde in PBS.

### 2.3 Culture of *ex vivo* skin and irradiation with red light

Fresh human skin (from Caucasian females, abdominal region, full thickness) was obtained from Biopredic International (Rennes, France). The protocol was approved by the ethics committee of Shiseido Mirai Technology Institute, and was in accordance with the National Institute of Health guideline and Declaration of Helsinki principles. The patients provided their informed consent to participate in this study. Skin samples were cut into 1.0 × 0.5 cm pieces, then irradiated with red light and cultured.^18^ On the following day, these day 1 samples were fixed with 4% paraformaldehyde in PBS. Other samples were similarly irradiated for 2 or 3 consecutive days, and then fixed in the same way.

### 2.4 Immunohistochemistry

The protocol for BrdU immunohistochemistry was described previously.^16^ Paraformaldehyde‐fixed samples were embedded in paraffin and sectioned at 3 μm. For BrdU and Ki‐67 staining, sections were deparaffinized and activated in HCl at 40°C or in Antigen Retrieval Buffer (100X Tris‐EDTA Buffer, pH 9.0) (ab93684, Abcam) at 90°C, then blocked with NGS blocking solution (10% normal goat serum and 0.4% Triton in 3% BSA/PBS) and stained with rat polyclonal anti‐BrdU [BU1/75 (ICR1)] antibody (1/700, ab6326, Abcam) or rabbit polyclonal anti‐Ki‐67 antibody (1/100, ab16667, Abcam). The secondary antibodies were rabbit anti‐rat IgG biotinylated antibody (1/200, BA4001, Abcam) or donkey anti‐rabbit 594 antibody. For BrdU staining, a VECTASTAIN ABC Standard kit (Vector Laboratories, Burlingame, USA), DAB substrate (Roche, Basel, Switzerland) and hematoxylin 3G (Sakura Finetek Japan, Japan, Tokyo) were used. For Ki‐67 staining, Prolong gold (Invitrogen) and NucBlue Fixed Cell Stain ReadyProbes DAPI (Invitrogen) were used. To evaluate BrdU‐ and Ki‐67‐positive cells, 5–6 images per section were acquired with a BX51/DP80 or BX53/DP74 fluorescence microscope (EVIDENT, Tokyo, Japan) using cellSens software (EVIDENT). The ratio of positive cells was determined by counting positive cells co‐stained with hematoxylin for BrdU and DAPI for Ki‐67 and dividing the result by the length of the insert in the epidermal‐equivalent model and the length of the basal membrane in human skin using ImageJ Fiji (National Institutes of Health).^19^ Average values were calculated.

### 2.5 Measurement of mitochondrial activity

An XF24 analyzer (Agilent Technologies, Santa Clara, CA, USA) was used to measure the mitochondrial oxygen consumption rate (OCR) and extracellular acidification rate (ECAR). Before the assay, the cartridge sensor was hydrated overnight in Seahorse Bioscience XF24 Calibration buffer (Agilent Technologies) at 37°C without CO_2_. On the day of the assay, the epidermal‐equivalent models were irradiated with red light (630 nm) for 2 h, then set on XF Islet Capture Microplates (Agilent Technologies), and XF DMEM Medium (Agilent Technologies) was added. The models were incubated at 37°C without CO_2_ for 1 h. OCR and ECAR were monitored under basal conditions and measured after injection of oligomycin (6 μM), FCCP (8 μM), antimycin A and rotenin (8 μM). The results were analyzed using Seahorse XF24 software.

### 2.6 Statistics

The results are expressed as the mean ± SD or the mean ± SE. The statistical significance of differences among the five groups was determined by ANOVA with Dunnet's test (Table 1) and the significance of differences between two groups was determined by applying Student's *t* test (Tables 2 and 4) or a paired‐*t* test (Table 5) as implemented in KaleidaGraph (HULINKS, Tokyo, Japan).

**TABLE 1:** Ratio of BrdU‐positive cells in the epidermal‐equivalent model after irradiation with red light.

Time (hour)	Intensity (J/cm^2^)	BrdU‐positive cells (%)
0	0	100.0 ± 17.8
0.5	11.16	112.0 ± 7.9
1	22.32	122.2 ± 17.5
2	44.64	135.3 ± 18.1^*^
3	66.96	130.8 ± 15.6^*^

The ratio of BrdU‐positive cells was determined after irradiation with red light for 0 h (control), 0.5 h, 1 h, 2 and 3 h. The untreated control is taken as 100%. ANOVA with Dunnet's test,

^*^ *p* < 0.05. Mean ± SD (*n* = 4).

**TABLE 2:** Ratio of Ki‐67‐positive cells in the epidermal‐equivalent model after irradiation with red light.

Time (hour)	Intensity (J/cm^2^)	Ki‐67‐positive cells (%)
0	0	100.0 ± 7.4
2	44.64	113.1 ± 5.9^*^

The ratio of Ki‐67‐positive cells was determined after irradiation with red light for 0 h (control) and 2 h. The untreated control is taken as 100%. Student's *t* test,

^*^ *p* < 0.05. Mean ± SD (*n* = 4).

**TABLE 3:** Ratio of BrdU‐ and Ki‐67‐positive cells in the epidermal‐equivalent model after irradiation with blue light.

Marker	Time (hour)	Intensity (J/cm^2^)	Positive cells (%)
BrdU	0	0	100.0 ± 24.0
	2	44.64	85.0 ± 21.4
Ki‐67	0	0	100.0 ± 21.1
	2	44.64	85.7 ± 6.5

The ratio of BrdU‐ and Ki‐67‐positive cells was determined after irradiation with blue light for 0 h (control) and 2 h. The untreated control is taken as 100%. Mean ± SD (*n* = 6).

## 3 RESULTS

**FIGURE 1:**
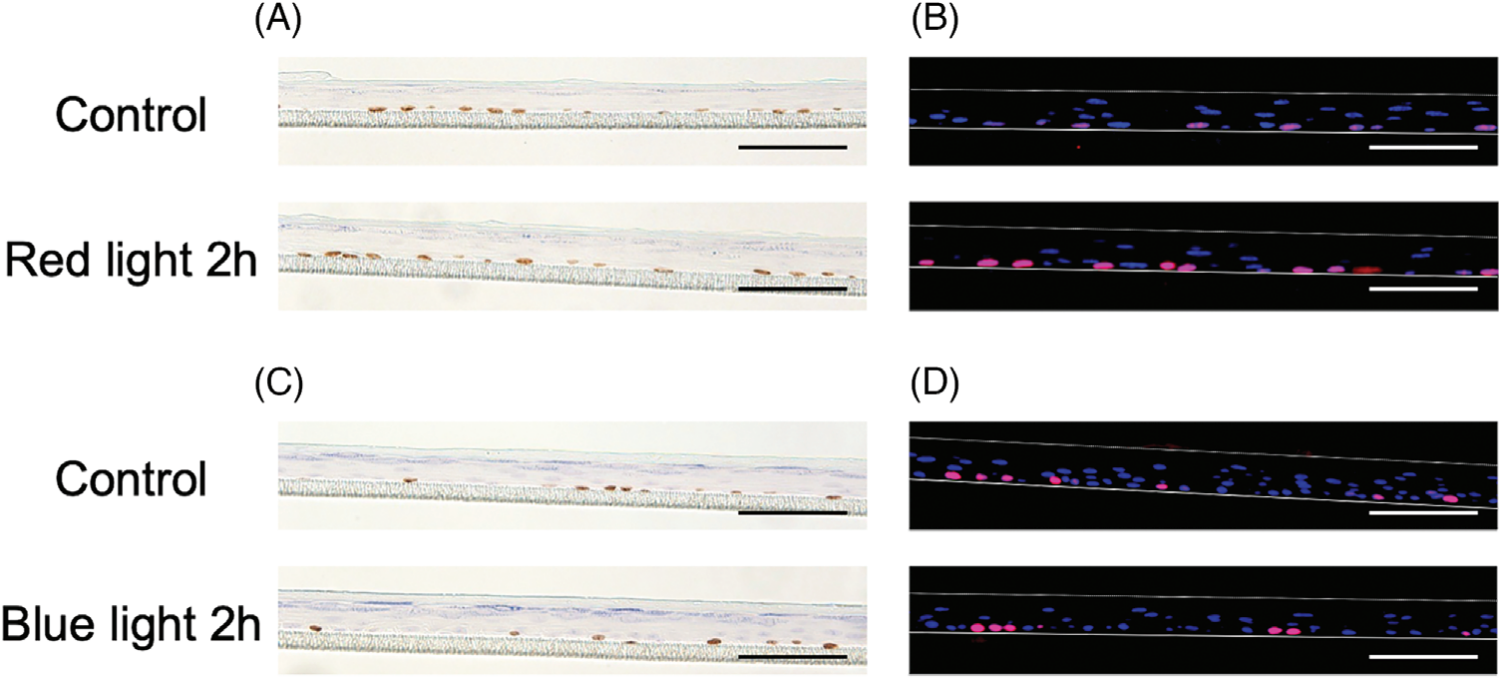
BrdU and Ki‐67 staining of epidermal‐equivalent models after irradiation with red and blue light. Representative images of (A) BrdU‐stained and (B) Ki‐67‐stained epidermal‐equivalent models after irradiation with red light for 0 h (control) and 2 h and (C) BrdU‐stained and (D) Ki‐67‐stained epidermal‐equivalent models after irradiation with blue light for 0 h (control) and 2 h. Scale bar 100 μm.

First, we performed immunohistochemistry of BrdU and Ki‐67 after irradiation of the epidermal‐equivalent model with red light. BrdU is a synthetic nucleoside analog of thymidine,^20^, ^21^, ^22^ which is incorporated in the S phase of the cell cycle and can be stained with BrdU antibody. Ki‐67 protein is a widely used proliferation marker^20^, ^21^, ^23^ that is expressed in the S, G1, G2, and M phases of the cell cycle, while it is absent in the G0 phase and in differentiated cells. The ratios of BrdU‐ and Ki‐67‐positive cells are shown in Tables 1 and 2, and representative images are shown in Figure 1A and B. Significant differences were found after irradiation with red light for 2 and 3 h. No significant difference was seen after irradiation with blue light (Table 3 and Figure 1C and D).

**FIGURE 2:**
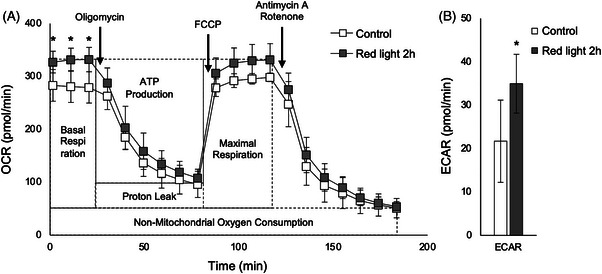
Measurement of OCR and ECAR in the epidermal‐equivalent model after irradiation with red light. (A) OCR profile and (B) ECAR. Student's *t* test, *p < 0.05. Mean ± SD (*n* = 6).

Based on the above results, we speculated that mitochondrial activity might be changed after irradiation with red light. The results of evaluation of mitochondrial activity using an XF24 analyzer are shown in Figure 2A and B, and the results of quantification of OCR and ECAR are shown in Table 4. Basal respiration and ATP production were significantly increased in the irradiated models, but maximal respiration was not changed. Consequently, EACR was higher in the irradiated models.

**TABLE 4:** Mitochondrial OCR and ECAR in the epidermal‐equivalent model after irradiation with red light.

		Control	Red light 2 h
OCR (pmol/min)	Basal respiration	228.2 ± 34.7	277.9 ± 23.1^*^
	ATP production	183.3 ± 28.7	224.2 ± 26.5^*^
	Proton leak	45.0 ± 12.7	53.7 ± 9.2
	Maximal respiration	247.2 ± 23.9	277.5 ± 28.7
	Spare respiratory capacity	19.0 ± 28.2	‐0.4 ± 18.9
	Non‐mitochondrial oxygen consumption	51.1 ± 18.3	54.1 ± 9.5
ECAR (pmol/min)		21.7 ± 9.5	34.7 ± 6.8^*^

Quantification of OCR parameters (basal respiration, ATP production, proton leak, maximal respiration, spare respiratory capacity, non‐mitochondrial OCR) and ECAR. Student's *t* test,

^*^ *p* < 0.05. Mean ± SD (*n* = 6).

**FIGURE 3:**
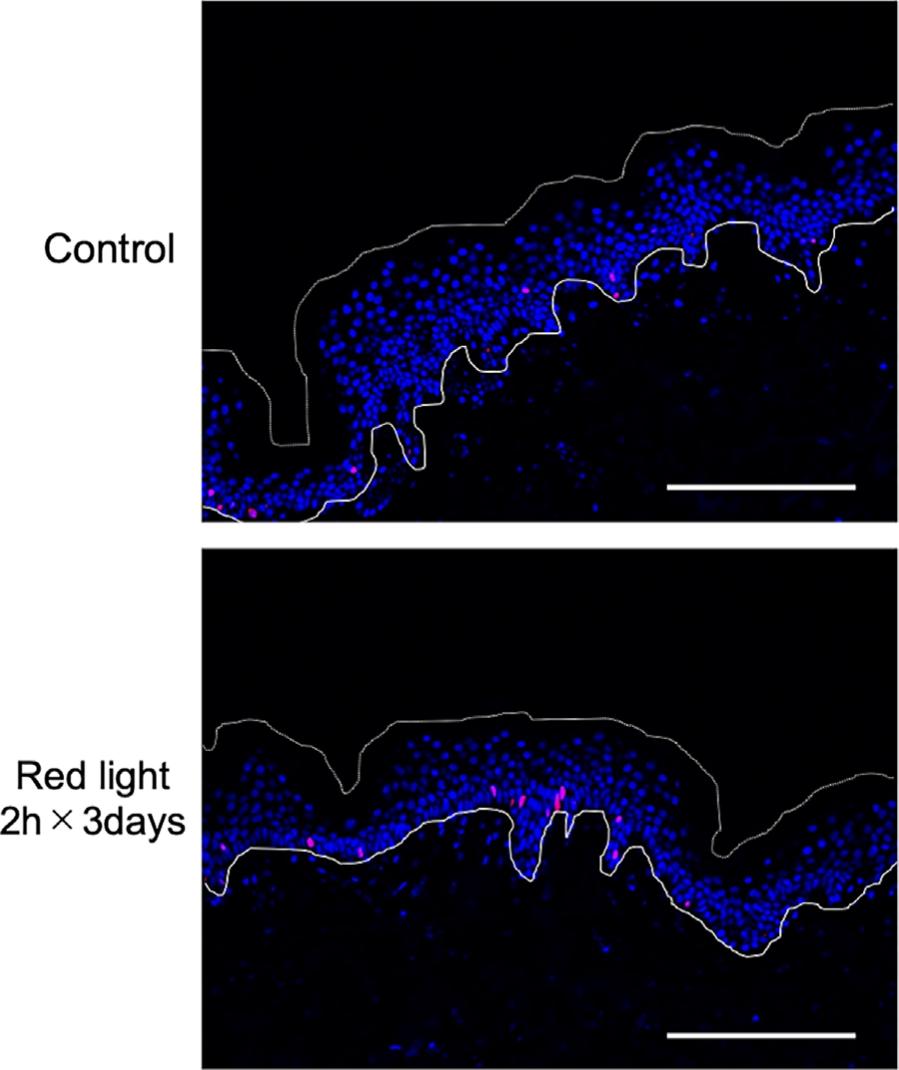
Representative images of human skin after irradiation with red light for 0 h (control) and 2 h for three consecutive days. Scale bar 200 μm.

Next, we performed immunohistochemistry of human skin tissue in culture. The results of Ki‐67 staining are shown in Table 5 and Figure 3. There was no change of epidermal proliferation after irradiation with red light for 1 day or 2 consecutive days (data not shown), but irradiation with red light for 2 h on three consecutive days significantly increased the ratio of Ki‐67‐positive cells. Thus, both the epidermal‐equivalent model and human skin showed increased keratinocyte proliferation after irradiation with red light.

**TABLE 5:** Ratio of Ki‐67‐positive cells in human skin after irradiation with red light.

Time (hour)	Intensity (J/cm^2^)	Ki‐67‐positive cells (%)
0	0	100.0 ± 42.5
2 h × 3 days	133.92 (44.64 J/cm^2^ × 3 days)	138.1 ± 50.3^*^

The ratio of Ki‐67‐positive cells after irradiation with red light for three consecutive days. The untreated control is taken as 100%. Paired‐*t* test,

^*^ *p* < 0.05. Mean ± SE (*n* = 5).

## 4 DISCUSSION

Our results show that cell proliferation in an epidermal‐equivalent model was increased after irradiation with red light for 2 h or 3 h, whereas irradiation with blue light of the same energy had no effect. In addition, mitochondrial activity was increased after irradiation with red light for 2 h. We used BrdU and Ki‐67 antibodies to evaluate proliferative activity. Incorporation of BrdU and expression of Ki‐67 are widely used as proliferation markers and are observed only in the stratum basale of the epidermis.^20^, ^21^ Keratinocytes in the basal layer of the epidermis are mainly responsible for proliferation, and loss of proliferation leads to impaired epidermal homeostasis.^17^ Therefore, maintaining basal proliferation is important.

OCR is used to measure oxidative phosphorylation and ECAR is used to measure glycolysis.^24^, ^25^ Our measurements indicated that basal respiration was increased by red light, but maximal respiration was not, suggesting that red light increases basal mitochondrial activity rather than spare capacity. It was previously reported that red light (30 J/cm^2^, 655 nm) increased the metabolic activity of keratinocytes, but blue light (477 nm) of the same energy did not.^11^ Further, exposure to red light (670 nm) increased mitochondrial membrane potential and cytochrome c oxidase (COX) expression in mice with age‐related macular degeneration.^26^ ATP was increased in association with increased expression of COX and reduced expression of acrolein, which is a marker of free radical‐induced retinal oxidative stress.^27^ These reports are consistent with the view that red light increases metabolic activity in the epidermis.

We also found that epidermal proliferation was increased in human skin after irradiation with red light for 2 h on three consecutive days. Proliferation markers decreased with increasing culture time in unirradiated skin,^28^ and red light might suppress this decrease.

The mortality of mitochondrial transcription factor A (TFAM) knockout mice is increased due to loss of epidermal barrier function and these mice also have defective oxidative phosphorylation and low ROS levels.^17^ Therefore, these mitochondrial functions might be required for keratinocyte proliferation, differentiation, and epidermal barrier homeostasis. In this research, we evaluated proliferation markers on the day after irradiation, and mitochondrial activity was measured immediately after irradiation with red light. Epidermal barrier recovery and lamellar granule secretion were both accelerated by red light within 1 h after the barrier disruption.^12^ On the other hand, red light exposure for 1 h did not significantly influence proliferation. The barrier recovery and increased mitochondrial activity might be related, but the details remain to be clarified. The effects of red light on epidermal proliferation and epidermal barrier recovery might involve different biochemical pathways.

Thus, our findings indicate that epidermal proliferation in both an epidermal‐equivalent model and human skin is increased by exposure to red light, in association with increased mitochondrial activity. These results suggest that exposure to red light may accelerate human epidermal proliferation and promote epidermal homeostasis.

## CONFLICT OF INTEREST STATEMENT

The authors declare that they have no conflict of interest.

## Supporting information

Supporting information

## Data Availability

The data that support the findings of this study are available from the corresponding author upon reasonable request.

## References

[1] Denda M , Nakanishi S . Do epidermal keratinocytes have sensory and information processing systems? Exp Dermatol. 2022;31:459‐474. 34726302 10.1111/exd.14494

[2] Matsumura Y , Ananthaswamy HN . Toxic effects of ultraviolet radiation on the skin. Toxicol Appl Pharmacol. 2004;195:298‐308. 15020192 10.1016/j.taap.2003.08.019

[3] Nakashima Y , Ohta S , Wolf AM . Blue light‐induced oxidative stress in live skin. Free Radic Biol Med. 2017;108:300‐310. 28315451 10.1016/j.freeradbiomed.2017.03.010

[4] Tsuchida K , Sakiyama N . Blue light‐induced lipid oxidation and the antioxidant property of hypotaurine: evaluation via measuring ultraweak photon emission. Photochem Photobiol Sci. 2023;22:345‐356. 36271182 10.1007/s43630-022-00319-8

[5] Shimizu S , Aoki A , Takahashi T , Harano F . Infrared‐A irradiation‐induced inhibition of human keratinocyte proliferation and potential mechanisms. Photochem Photobiol. 2020;96:1105‐1115. 32118302 10.1111/php.13248 PMC7586992

[6] Hudson L , Rashdan E , Bonn CA , Chavan B , Rawlings D , Birch‐Machin MA . Individual and combined effects of the infrared, visible, and ultraviolet light components of solar radiation on damage biomarkers in human skin cells. FASEB J. 2020;34:3874‐3883. 31944399 10.1096/fj.201902351RR PMC7079185

[7] Cho S , Lee MJ , Kim MS , et al. Infrared plus visible light and heat from natural sunlight participate in the expression of MMPs and type I procollagen as well as infiltration of inflammatory cell in human skin in vivo. J Dermatol Sci. 2008;50:123‐133. 18194849 10.1016/j.jdermsci.2007.11.009

[8] de Abreu PTR , de Arruda JAA , Mesquita RA , Abreu LG , Diniz IMA , Silva TA . Photobiomodulation effects on keratinocytes cultured in vitro: a critical review. Lasers Med Sci. 2019;34:1725‐1734. 31154598 10.1007/s10103-019-02813-5

[9] Sheen YS , Fan SMY , Chan CC , Wu YF , Jee SH , Lin SJ . Visible red light enhances physiological anagen entry in vivo and has direct and indirect stimulative effects in vitro. Lasers Surg Med. 2015;47:50‐59. 25557083 10.1002/lsm.22316

[10] Gagnon D , Gibson TW , Singh A , zur Linden AR , Kazienko JE , LaMarre J . An in vitro method to test the safety and efficacy of low‐level laser therapy (LLLT) in the healing of a canine skin model. BMC Vet Res. 2016;12:73. 27056043 10.1186/s12917-016-0689-5 PMC4825076

[11] Castellano‐Pellicena I , Uzunbajakava NE , Mignon C , Raafs B , Botchkarev VA , Thornton MJ . Does blue light restore human epidermal barrier function via activation of Opsin during cutaneous wound healing? Lasers Surg Med. 2019;51:370‐382. 30168605 10.1002/lsm.23015

[12] Denda M , Fuziwara S . Visible radiation affects epidermal permeability barrier recovery: selective effects of red and blue light. J Invest Dermatol. 2008;128:1335–1336. 18007581 10.1038/sj.jid.5701168

[13] Abe Y , Konno H , Yoshida S , et al. Red light‐promoted skin barrier recovery: Spatiotemporal evaluation by transepidermal potential. PLoS One. 2019;14:e0219198. 31291308 10.1371/journal.pone.0219198 PMC6620005

[14] Umino Y , Ipponjima S , Denda M . Modulation of lipid fluidity likely contributes to the fructose/xylitol‐induced acceleration of epidermal permeability barrier recovery. Arch Dermatol Res. 2019;311:317‐324. 30847563 10.1007/s00403-019-01905-0

[15] Umino Y , Ipponjima S , Denda M . Polyoxyethylene/polyoxypropylene dimethyl ether (EPDME) random copolymer improves lipid structural ordering in stratum corneum of an epidermal‐equivalent model as seen by two‐photon microscopy. Skin Res Technol. 2021;27:632‐638. 33410546 10.1111/srt.12996

[16] Kumamoto J , Nakanishi S , Makita M , et al. Mathematical‐model‐guided development of full‐thickness epidermal equivalent. Sci Rep. 2018;8:17999. 30573749 10.1038/s41598-018-36647-y PMC6301960

[17] Sreedhar A , Aguilera‐Aguirre L , Singh KK . Mitochondria in skin health, aging, and disease. Cell Death Dis. 2020;11:444. 32518230 10.1038/s41419-020-2649-z PMC7283348

[18] Iriyama S , Yasuda M , Nishikawa S , Takai E , Hosoi J , Amano S . Decrease of laminin‐511 in the basement membrane due to photoaging reduces epidermal stem/progenitor cells. Sci Rep. 2020;10:12592. 32724130 10.1038/s41598-020-69558-y PMC7387558

[19] Schindelin J , Arganda‐Carreras I , Frise E , et al. Fiji: an open source platform for biological‐image analysis. Nat Methods. 2012;9:676‐682 22743772 10.1038/nmeth.2019 PMC3855844

[20] Onuma H , Mastui C , Morohashi M . Quantitative analysis of the proliferation of epidermal cells using a human skin organ culture system and the effect of DbcAMP using markers of proliferation (BrdU, Ki‐67, PCNA). Arch Dermatol Res. 2001;293:133‐138. 11357227 10.1007/s004030000195

[21] Tanaka R , Tainaka M , Ota T , et al. Accurate determination of S‐phase fraction in proliferative cells by dual fluorescence and peroxidase immunohistochemistry with 5‐bromo‐2'‐deoxyuridine (BrdU) and Ki67 antibodies. J Histochem Cytochem. 2011;59:791‐798. 21551319 10.1369/0022155411411090 PMC3261604

[22] Gartzner HG . Monoclonal antibody to 5‐bromo and 5‐iododeoxyuridine: a new reagent for detection of DNA replication. Science. 1982;218:474–475. 7123245 10.1126/science.7123245

[23] Gerdes J , Lelle RJ , Pickartz H , et al. Growth fractions in breast cancers determined in situ with monoclonal antibody Ki‐67. J Clin Pathol. 1986;39:977‐980. 3020096 10.1136/jcp.39.9.977 PMC500196

[24] Ferrick DA , Neilson A , Beeson C . Advances in measuring cellular bioenergetics using extracellular flux. Drug Discov Today. 2008;13:268‐274. 18342804 10.1016/j.drudis.2007.12.008

[25] Forni MF , Chausse B , Peloggia J , Kowaltowski AJ . Bioenergetic profiling in the skin. Exp Dermatol. 2016;25:147‐148. 26343263 10.1111/exd.12856

[26] Begum R , Powner MB , Hudson N , Hogg C , Jeffery G . Treatment with 670 nm light up regulates cytochrome C oxidase expression and reduces inflammation in an age‐related macular degeneration model. PLoS One. 2013;8:e57828. 23469078 10.1371/journal.pone.0057828 PMC3585189

[27] Gkotsi D , Begum R , Salt T , et al. Recharging mitochondrial batteries in old eyes. Near infra‐red increases ATP. Exp Eye Res. 2014;122:50‐53. 24631333 10.1016/j.exer.2014.02.023

[28] Boyce ST , Supp AP , Swope VB , Warden GD . Vitamin C regulates keratinocyte viability, epidermal barrier, and basement membrane in vitro, and reduces wound contraction after grafting of cultured skin substitutes. J Invest Dermatol. 2002;11:565‐572. 10.1046/j.1523-1747.2002.01717.x 11918700

