# Supplementary material for: Effect of red light on epidermal proliferation and mitochondrial activity
Source: Skin Res Technol. 2023 Aug 28;29(9):e13447. doi: 10.1111/srt.13447 (PMC10462800; doi:10.1111/srt.13447)
Supplement: Supplementary file 1 — Supporting information [file SRT-29-e13447-s001.docx]

**Supporting information**

**Fig. S1. Spectra of red and blue LEDs.**


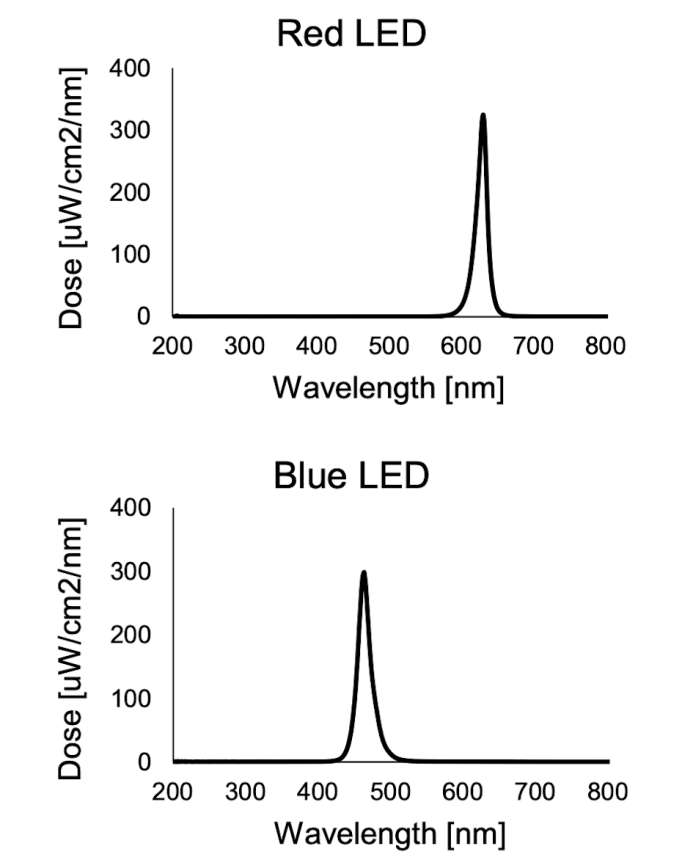


Red LED peak wavelength is 630 nm and half bandwidth is 17 nm. Blue LED peak wavelength is 463 nm and half bandwidth is 20 nm.
